# DDX5/METTL3-METTL14/YTHDF2 Axis Regulates Replication of Influenza A Virus

**DOI:** 10.1128/spectrum.01098-22

**Published:** 2022-05-18

**Authors:** Lingcai Zhao, Yongzhen Zhao, Qingzheng Liu, Jingjin Huang, Yuanlu Lu, Jihui Ping

**Affiliations:** a MOE Joint International Research Laboratory of Animal Health and Food Safety, Engineering Laboratory of Animal Immunity of Jiangsu Province, College of Veterinary Medicine, Nanjing Agricultural University, Nanjing, China; Regional Centre for Biotechnology

**Keywords:** DDX5, IFN-β, METTL3-METTL14/YTHDF2, antiviral immunity, influenza viruses

## Abstract

DEAD-box helicase 5 (DDX5), a member of the DEAD/H-box helicases, is known to participate in all aspects of RNA metabolism. However, its regulatory effect in antiviral innate immunity during replication of influenza virus remains unclear. Herein, we found that human DDX5 promotes replication of influenza virus in A549 cells. Moreover, our results further revealed that DDX5 relies on its N terminus to interact with the nucleoprotein (NP) of influenza virus, which is independent of RNA. Of course, we also observed colocalization of DDX5 with NP in the context of transfection or infection. However, influenza virus infection had no significant effect on the protein expression and nucleocytoplasmic distribution of DDX5. Importantly, we found that DDX5 suppresses antiviral innate immunity induced by influenza virus infection. Mechanistically, DDX5 downregulated the mRNA levels of interferon beta (IFN-β), interleukin 6 (IL-6), and DHX58 via the METTL3-METTL14/YTHDF2 axis. We revealed that DDX5 bound antiviral transcripts and regulated immune responses through YTHDF2-dependent mRNA decay. Taken together, our data demonstrate that the DDX5/METTL3-METTL14/YTHDF2 axis regulates the replication of influenza A virus.

**IMPORTANCE** The replication and transcription of influenza virus depends on the participation of many host factors in cells. Exploring the relationship between viruses and host factors will help us fully understand the characteristics and pathogenic mechanisms of influenza viruses. In this study, we showed that DDX5 interacted with the NP of influenza virus. We demonstrated that DDX5 downregulated the expression of IFN-β and IL-6 and the transcription of antiviral genes downstream from IFN-β in influenza virus-infected A549 cells. Additionally, DDX5 downregulated the mRNA levels of antiviral transcripts via the METTL3-METTL14/YTHDF2 axis. Our findings provide a novel perspective to understand the mechanism by which DDX5 regulates antiviral immunity.

## INTRODUCTION

From the severe acute respiratory syndrome (SARS) epidemic in Guangdong caused by the SARS coronavirus (CoV) in 2002 to the H1N1 influenza pandemic in 2009, the human infections with avian influenza H7N9 in China in the spring of 2013, the Ebola virus epidemics in West Africa in 2014 and then in December 2019, and the pneumonia epidemic caused by the new coronavirus (coronavirus disease 2019 [COVID-19]) in Wuhan City, Hubei Province, China (1–3), it can be seen that viral infectious pathogens can cause very high human infection rates and mortality and bring huge burdens to the global economy and human health.

Influenza viruses, with a wide host range, are some of the most destructive pathogens that threaten the poultry industry and general public health (4, 5). Influenza viruses harness gene expression mechanisms and metabolites of the host cell to replicate by interacting with several cellular proteins (6–8). Therefore, elucidating the interaction of influenza virus proteins with cellular proteins has great importance for the understanding of viral pathogenesis. RNA helicases are divided into two families, DExD-box helicases (DDX) and DExH-box helicases (DHX), which bear the Asp-Glu-X-Asp/His signature (9, 10). DExD/H-box helicases play critical roles in multiple cellular processes, including transcription, cellular RNA metabolism, translation, and infection (11, 12). Several studies in recent years reported that DEAD/H-box helicases participate in antiviral innate immunity, either by acting as viral nucleic acid sensors or by regulating downstream signaling events (9, 13, 14). DEAD-box helicase 1 (DDX1) stimulated interferon beta (IFN-β) activation in foot-and-mouth disease virus (FMDV)-infected cells, and chicken DDX1 acts as an RNA sensor to mediate IFN-β signaling pathway activation in antiviral innate immunity (15, 16). DDX3 directly facilitates IκB kinase subunit alpha (IKKα) activation, and DHX9 is critical to the activation of NF-κB and to the production of tumor necrosis factor alpha (TNF-α) and interleukin 6 (IL-6) (17, 18). DHX29 binds directly to nucleic acids and interacts with retinoic acid-inducible gene I (RIG-I) and mitochondrial antiviral-signaling protein (MAVS), activating the RIG-I–MAVS-dependent cytosolic nucleic acid response (19). DDX43 recruits TRIF or IPS-1 as an adaptor and activates the IFN-β pathway in Nile tilapia (20). Importantly, DEAD/H-box helicases also have complex interactions in cellular immune regulation. DDX1, DDX21, and DHX36 represent a double-stranded RNA (dsRNA) sensor that uses the TRIF pathway to activate type I IFN responses (21). However, some members of the DExD/H-box RNA helicases play a negative regulatory role in antiviral responses. DDX24 hijacked adaptor proteins FADD and RIP1 in host cells to suppress viral RNA-dependent interferon production (22). DDX46 inhibits antiviral innate responses by entrapping selected antiviral transcripts in the nucleus (23). DEAD-box helicase 5 (DDX5) is important in transcriptional regulation, which is hijacked by diverse viruses to facilitate viral replication. However, the roles of DDX5 in the replication of influenza virus are still unclear, and how human DDX5 modulates innate immune signaling is not fully understood.

The innate immune system is the first line of host defense against viral infection. Viral nucleic acids present as pathogen-associated molecular patterns (PAMPs), which are detected by pattern recognition receptors (PRRs) (24). The negative-sense RNA genome of influenza A virus (IAV) is recognized by RIG-I-like receptors (RLRs), mainly RIG-I (DDX58) (25, 26). Upon recognition of viral RNA, RIG-I undergoes conformational changes and interacts with the mitochondrial adaptor protein MAVS (27, 28), a central platform for TRAF3 and TRAF6 engagement, which is followed by the activation of TANK-binding kinase 1 (TBK1)/IKKε and transforming growth factor β (TGF-β)-activated kinase 1 (TAK1)/IKK kinases, the subsequent phosphorylation of IFN regulatory factor 3 (IRF3) and NF-κB, and the ultimate induction of type I interferons and downstream antiviral genes (29–32). Type I IFN (IFN-α and -β) induces the expression of hundreds of interferon-stimulated genes (ISGs) and eventually an antiviral state for host cells (33). In recent years, studies have reported that some members of the DDX family regulate antiviral innate immune responses through RNA modification. DDX46 bound Mavs, Traf3 and Traf6 transcripts via their conserved CCGGUU motifs and therefore prevented their translation (23). DDX5 regulates *N*^6^-methyladenosine (m6A) levels on the DHX58 and NF-κB transcripts to dampen antiviral innate immunity (34). However, whether human DDX5 participates in regulating the production of type I interferons, especially the m6A modification in regulating type I interferons, is unknown.

m6A is the most prevalent modification on eukaryotic mRNA and plays an important role in regulating multiple biological processes. m6A modification is a dynamic and reversible regulatory process, which is composed of “writers,” “erasers,” and “readers.” m6A writers consist of the catalytic subunit METTL3 and cofactors like METTL14 and WTAP (35–37). m6A erasers, including FTO and ALKBH5, erase m6A modification and play reversible regulatory roles. The modification is then functionally interpreted through the binding of m6A readers. The cytoplasmic YTH domain family 1 (YTHDF1), YTHDF2, and YTHDF3 proteins have been shown to directly bind and recognize m6A (38, 39), mediating myriad cellular processes, including mRNA decay and translation efficiency (40).

The precise roles of DDX5, apparently a multifaceted protein (41), in the replication of influenza viruses remain largely obscure. Here, we reveal that DDX5 interacts with the nucleoprotein (NP) of influenza virus. Importantly, DDX5 is involved in the regulation of the immune response induced by influenza virus infection in A549 cells, especially the type I interferon signaling pathway. We elucidated that the DDX5/METTL3-METTL14/YTHDF2 signaling axis regulated antiviral transcripts, including mRNAs of IFN-β, IL-6, and DHX58. Together, our findings add insight into the action of the DDX5/METTL3-METTL14/YTHDF2 signaling axis in regulating the innate response to influenza virus infection.

## RESULTS

### Multiple DExH-box helicases regulate the replication of influenza virus.

**FIG 1:**
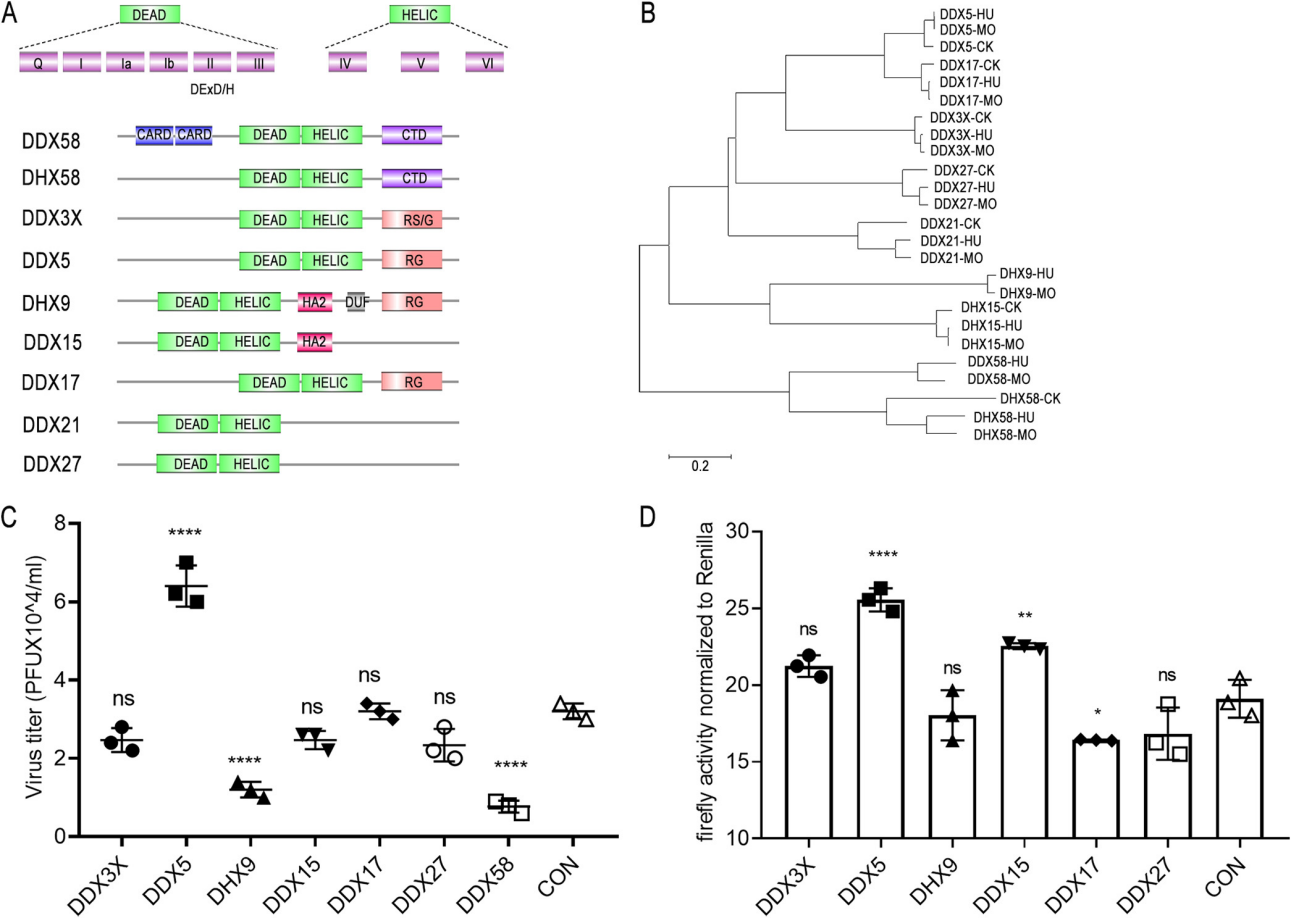
Multiple DExH-box helicases regulate replication of influenza virus. (A) Protein domain schematic for representatives of DEAD/H-box helicases. DExD/H-box helicases contain conserved helicase domains 1 and 2, which consist of motifs I to VI. DExD/H-box is located within motif II, and the HELIC domain is a conserved C-terminal helicase domain. CARD, caspase activation and recruitment domain; CTD, C-terminal domain; RG, arginine- and glycine-rich domain; RS/G, arginine- and serine-/glycine-rich domain; HA2, helicase-associated domain 2; DUF, domain of unknown function. (B) A neighbor-joining phylogenetic tree of DEAD/H-box helicases from different host sources was generated with MEGA6 software. (C) Plasmid encoding DDX3X, DDX5, DHX9, DDX15, DDX17, DDX27, or DDX58 or empty pCAGGS vector was transfected into A549 cells, and 24 h after transfection, cells were infected with WSN/1933 at an MOI of 0.05. The supernatants were sampled at 24 h after infection, and the virus titers were determined by plaque assays. The results are shown as average values from 3 replicates ± standard deviations. (D) Polymerase gene expression plasmids expressing poll-Luc and RL-TK and corresponding plasmids encoding DExD/H-box helicases or empty pCAGGS vector were cotransfected into HEK293T cells, and 48 h after transfection, cells were lysed, the protein lysate was harvested, and the polymerase activity was detected. Reconstructed polymerases were from influenza virus H1N1 (A/WSN/1933). The data indicate the firefly luciferase activity normalized to the *Renilla* luciferase activity. Statistical differences between groups are labeled according to one-way ANOVA followed by Dunnett’s test. Each treatment was repeated three times in parallel. The results are presented as mean values ± standard deviations. *, *P* < 0.05; **, *P* < 0.01; ****, *P* < 0.0001; ns, no significance.

DEAD/H-box RNA helicases were initially described as key factors involved in multiple cellular processes of RNA metabolism. Interestingly, these helicases have antiviral and proviral functions and behave as attractive targets between host innate immune systems and viruses. There is evidence that many DExD/H-box helicases, such as DDX39B, DDX3, DDX56 (42, 43), etc., have also been identified as essential host factors for the replication of different viruses. However, many questions persist about their relations with intrinsic immune processes and viruses. Double-stranded RNA can be detected by the cytoplasmic RNA helicase proteins RIG-I (DDX58) and MDA5, two proteins that share sequence similarities within a caspase recruitment domain (CARD) and a DExD/H-box RNA helicase domain. Another helicase protein, LGP2 (DHX58), lacks the CARD region and does not activate IFN-β gene expression (Fig. 1A). Phylogenetic-tree analyses found that DDX proteins in mammals are relatively closely related; however, they are farther from avian origins (Fig. 1B). Interestingly, several pairs of DDX proteins with high homology belonged to the same subclade, including DDX5 and DDX17, DHX9 and DHX15, and DDX58 and DHX58. They may have similar functions or play complementary roles.

To understand the roles of DExH-box helicases in the replication of influenza A virus, we screened multiple DExH-box helicases, including DDX3X, DDX5, DHX9, DDX15, DDX17, DDX27, and DDX58. First, we constructed the corresponding protein expression plasmids. Next, we sought to investigate the impact of these DExH-box helicases on the replication of influenza A viruses in human cells. A549 cells transfected with the corresponding protein expression plasmids were infected with WSN/1933, and virus replication was monitored at 24 h postinfection. The results showed that several DExH-box helicases regulated the replication of influenza virus. DDX5 positively regulated influenza virus replication, whereas DDX58 and DHX9 negatively regulated influenza virus replication (Fig. 1C). DDX58 is known to inhibit influenza A virus replication. DHX9, a DExDc helicase family member, functions as an important viral dsRNA sensor in myeloid dendritic cells. Next, we tested the effect of DExH-box helicases on viral RNA synthesis as measured in the dual-luciferase minigenome assay. The results showed that overexpression of DDX5 slightly affected the polymerase activity of influenza virus (Fig. 1D). However, the regulatory mechanism of DDX5 in influenza virus replication has not been reported.

### DDX5 promotes multiple rounds of influenza virus replication.

**FIG 2:**
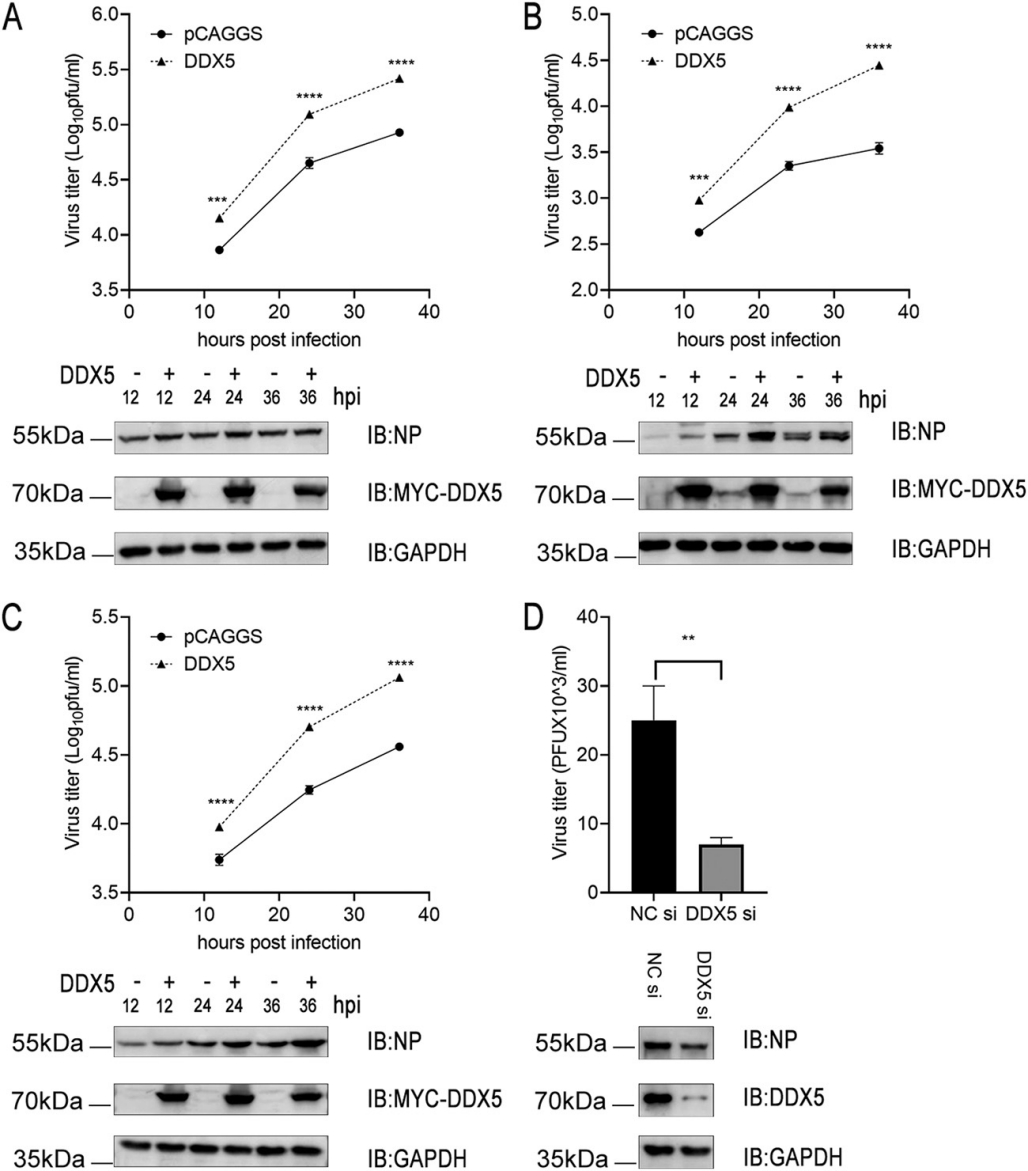
DDX5 promotes multiple rounds of influenza virus replication. (A, B, and C) A549 cells were transfected with MYC-DDX5 or empty pCAGGS vector. At 24 h after transfection, cells were infected with WSN-H7 (MOI = 0.01) (A), WSN/1933 (MOI = 0.01) (B), or H9N2 (MOI = 0.01) (C) virus, respectively, and the supernatants were sampled at 12 h, 24 h, and 36 h after infection. The viral NP expression levels were measured by Western blotting, and viral titers in the supernatants were determined by plaque assay. (D) A549 cells were transfected with siRNA targeting DDX5 or with scrambled siRNA for 24 h. The cells were infected with WSN-H7 (MOI = 0.05) and cultured for 24 h. The viral NP expression levels were measured by Western blotting, and viral titers were measured by plaque assay. Each treatment was repeated three times in parallel, and the results are shown as the average values from 3 replicates ± standard deviations. **, *P* < 0.01; ***, *P* < 0.001; ****, *P* < 0.0001.

To further understand the function of the aforementioned DExH-box helicases in viral replication, we performed Gene Ontology (GO) functional annotation and KEGG pathway enrichment analysis (Table S1 in the supplemental material). The results showed that these DExH-box helicases were significantly enriched in terms related to spliceosome, ribonucleoprotein complex biogenesis, RNA secondary structure unwinding, mRNA transport, and novel intracellular components of the RIG-I-like receptor (RLR) pathway (Fig. S1A). To further capture the relationships between these DExH-box helicases, a network of protein-protein interactions was constructed. We identified densely connected network components, including DDX5, DDX17, DHX15, and DHX9 (Fig. S1B). DDX5, also called p68, has been demonstrated to regulate the replication of various viruses (44). Thus, we aimed to determine the impact of DDX5 on the infection of A549 cells with influenza A virus. We found that overexpression of DDX5 promoted replication and protein expression of influenza viruses, including WSN-H7, WSN/1933, and H9N2 viruses (Fig. 2A to C). To elucidate the mechanisms of DDX5-mediated effects on viral replication, we knocked down the protein expression of DDX5 by small interfering RNA (siRNA)-mediated silencing. The results showed that knockdown of DDX5 was not conducive to influenza virus replication (Fig. 2D).

### DDX5 interacts with NP of influenza virus.

**FIG 3:**
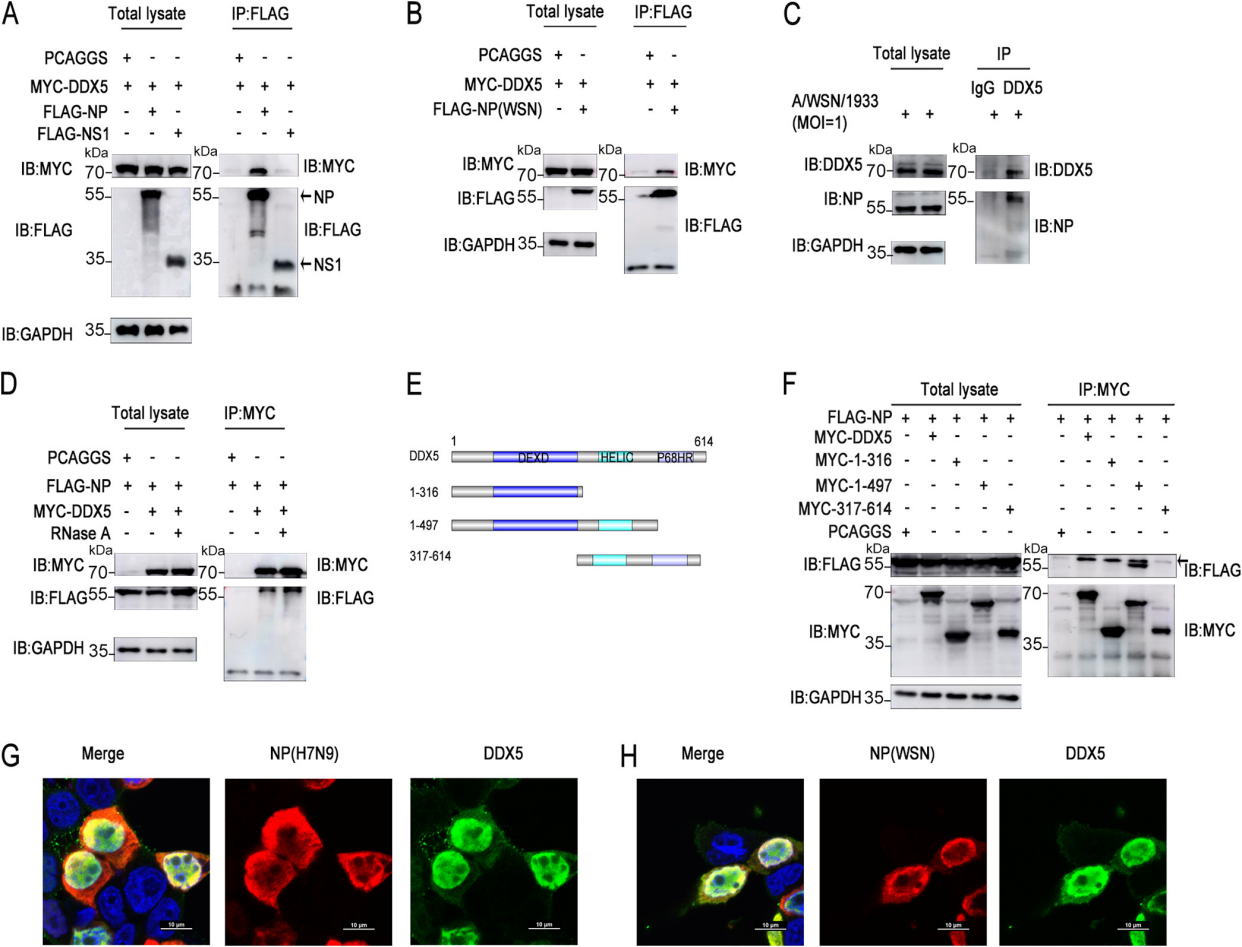
DDX5 interacts with the NP protein of influenza virus. (A) HEK293T cells were cotransfected with MYC-DDX5 and FLAG-NP or FLAG-NS1 of influenza virus A/Anhui/1/2013 (H7N9). The cells were lysed to harvest the protein lysate at 48 h after transfection, followed by co-IP with anti-FLAG mouse MAb and Western blotting using anti-MYC mouse MAb and anti-FLAG mouse MAb. (B) HEK293T cells were cotransfected with MYC-DDX5 and FLAG-NP of influenza virus A/WSN/1933 (H1N1). The cells were lysed to harvest the protein lysate at 48 h after transfection, followed by co-IP with anti-FLAG mouse MAb and Western blotting using anti-MYC mouse MAb and anti-FLAG mouse MAb. (C) A549 cells were infected with WSN/1933 (MOI = 1) and cultured for 12 h, and then the cells were lysed for co-IP experiments with anti-IgG mouse MAb or anti-DDX5 mouse MAb and Western blotting using anti-DDX5 mouse MAb and anti-NP mouse MAb. (D) HEK293T cells were cotransfected with MYC-DDX5 and FLAG-NP, and at 48 h after transfection, the cells were lysed and cell lysates were treated with or without RNase A, followed by co-IP with anti-MYC mouse MAb. (E) Schematic of full-length DDX5 and its truncated mutants. (F) HEK293T cells were cotransfected with FLAG-NP and full-length DDX5 or its truncated mutants. The cells were lysed to harvest the protein lysate at 48 h after transfection, followed by co-IP with anti-MYC mouse MAb and Western blotting using anti-MYC mouse MAb and anti-FLAG mouse MAb. Horseradish peroxidase (HRP)-labeled goat anti-mouse light IgG light chain as secondary antibody for eliminating heavy chain interference was used in the above-described co-IP experiments. (G and H) MYC-DDX5 plasmids were transfected together with expression plasmids encoding FLAG-NP protein of influenza virus A/Anhui/1/2013 (H7N9) (G) or A/WSN/1933 (H1N1) (H) into HEK293T cells, and 24 h after transfection, the cells were fixed, permeabilized, and stained with anti-MYC and anti-FLAG antibodies, followed by immunostaining with A546-labeled goat anti-mouse secondary antibody or FITC-labeled goat anti-rabbit secondary antibody. Then, the cells were stained with DAPI and examined via fluorescence microscopy.

Based on previous studies on the interaction of DEAD/H-box RNA helicases with influenza virus proteins, we found that DEAD/H-box RNA helicases were more likely to bind to NP and nonstructural protein 1 (NS1) of influenza virus (Table S2). To explain the molecular mechanism of the regulation of influenza virus replication by DDX5, we further explored DDX5-interacting viral proteins. Coimmunoprecipitation (co-IP) assays showed that DDX5 interacted with NP of influenza virus, but not with NS1 (Fig. 3A). Next, we found that DDX5 also interacted with NP of influenza virus WSN/1933 (Fig. 3B). Of course, we also detected the interaction of endogenous DDX5 with NP in influenza virus-infected cells (Fig. 3C). To test the possibility that the association was mediated by nucleic acids, RNase A treatment in cell lysates was used, and it did not affect the interaction of DDX5 with NP (Fig. 3D), establishing that this association was not mediated by single-stranded RNA (ssRNA). To determine which DDX5 domain interacts with NP, we constructed DDX5 mutants (Fig. 3E). Compared with other mutants, DDX5 with amino acids 1 to 316 deleted lost the capacity to interact with NP, suggesting that the DEXD domain may play an important role in the interaction between DDX5 and NP (Fig. 3F). As anticipated, it was observed that DDX5 and NP colocalized widely in and around the nucleus (Fig. 3G and H).

### Infection by influenza virus has no significant effect on the protein expression and cellular sublocalization of DDX5.

**FIG 4:**
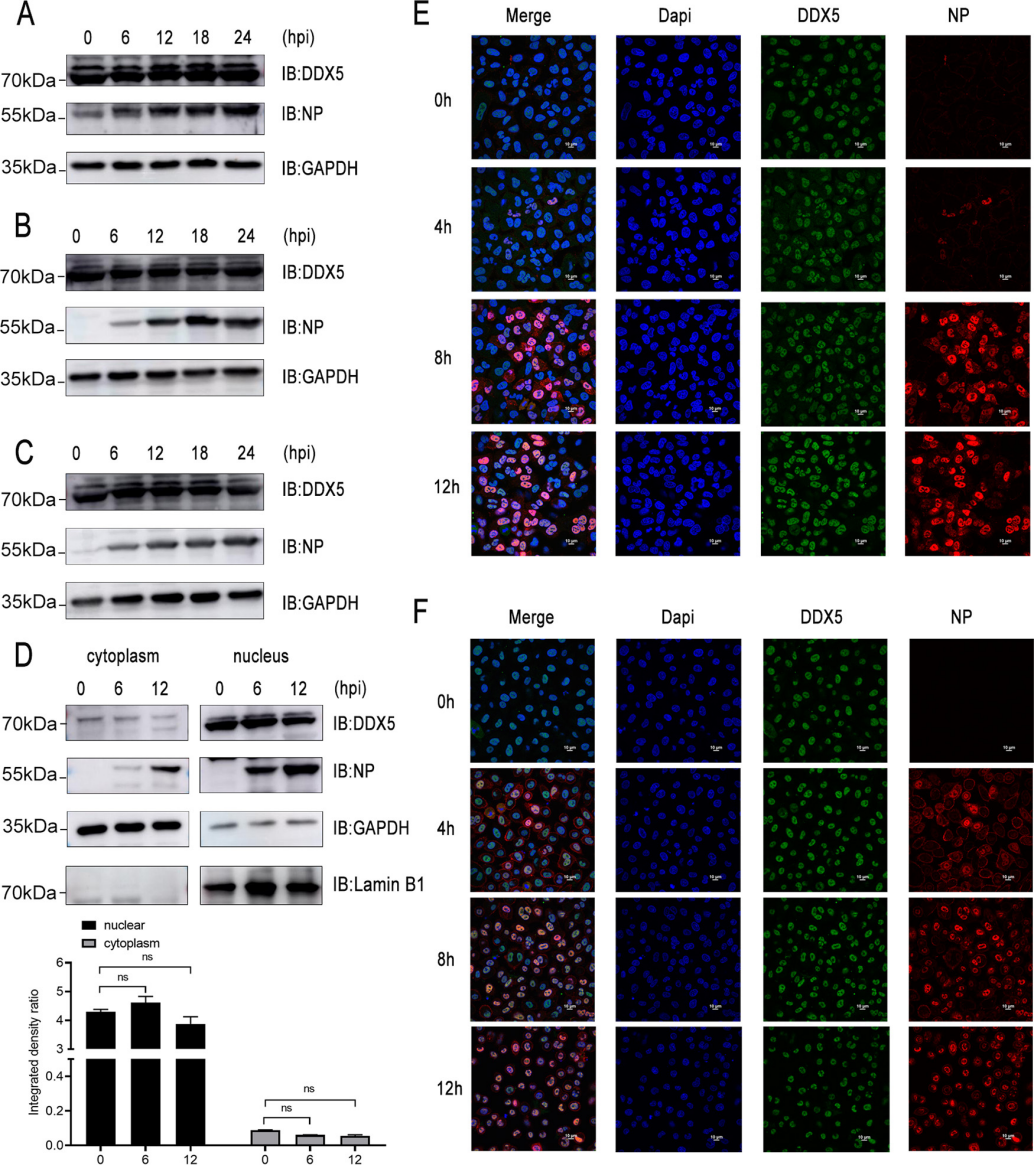
Infection with influenza virus has no significant effect on the protein expression and cellular sublocalization of DDX5. (A, B, and C) A549 cells were infected with influenza virus WSN-H7 (A), WSN/1933 (B), or H9N2 (C) at an MOI of 0.5. At 0 h, 6 h, 12 h, 18 h, and 24 h after infection, cells were lysed to harvest the protein lysate, and protein expression levels were detected by Western blotting. (D) A549 cells were infected with influenza virus WSN/1933 at an MOI of 1. At 0 h, 6 h, and 12 h after infection, nuclear and cytoplasmic proteins were isolated separately. Protein expression levels were detected by Western blotting. The expression levels of viral NP protein and DDX5 were normalized to GAPDH expression using ImageJ. ns, no significance. (E and F) A549 cells were infected with influenza virus WSN/1933 (E) or recombinant influenza virus WSN-H7 (F) at an MOI of 0.5. At 0 h, 4 h, 8 h, and 12 h after infection, cells were fixed, permeabilized, and stained with anti-DDX5 antibody and anti-NP antibody, followed by immunostaining with A546-labeled goat anti-mouse secondary antibody or FITC-labeled goat anti-rabbit secondary antibody. Then, cells were stained with DAPI and examined via fluorescence microscopy.

A previous study showed that vesicular stomatitis virus (VSV) and herpes simplex virus (HSV) infections induced upregulation of DDX5 expression in a time-dependent manner in peritoneal macrophages (45). We were interested to know whether DDX5 protein levels were altered during influenza virus infection. However, we found that infections with recombinant influenza virus WSN-H7 (Fig. 4A), human influenza virus WSN/1933 (Fig. 4B), and avian influenza virus H9N2 (Fig. 4C) had no significant effect on the expression of DDX5 in A549 cells. We then analyzed whether influenza virus infection alters the cellular localization of DDX5. We detected the protein levels of DDX5 in the nucleus and cytoplasm of A549 cells during influenza virus infection. The results showed that NP of influenza virus and intracellular DDX5 protein were mainly distributed in the nucleus and that influenza virus infection had no significant effect on the nucleocytoplasmic distribution of DDX5 (Fig. 4D). To further observe the nucleoplasmic distribution of DDX5 during influenza virus infection, we performed immunofluorescence experiments. The results showed that NP protein and DDX5 colocalized extensively mainly in the nucleus of A549 cells infected with influenza virus WSN/1933 (Fig. 4E) or recombinant influenza virus WSN-H7 (Fig. 4F). In addition, DDX5 was mainly distributed in the nucleus during the process of influenza virus infection, and there was no obvious change. In general, unlike other RNA viruses and DNA viruses, infection by influenza A viruses did not cause changes in the protein level and nucleocytoplasmic distribution of DDX5 in A549 cells.

### Overexpression or knockdown of DDX5 affected IAV-triggered IFN-β and ISG production.

**FIG 5:**
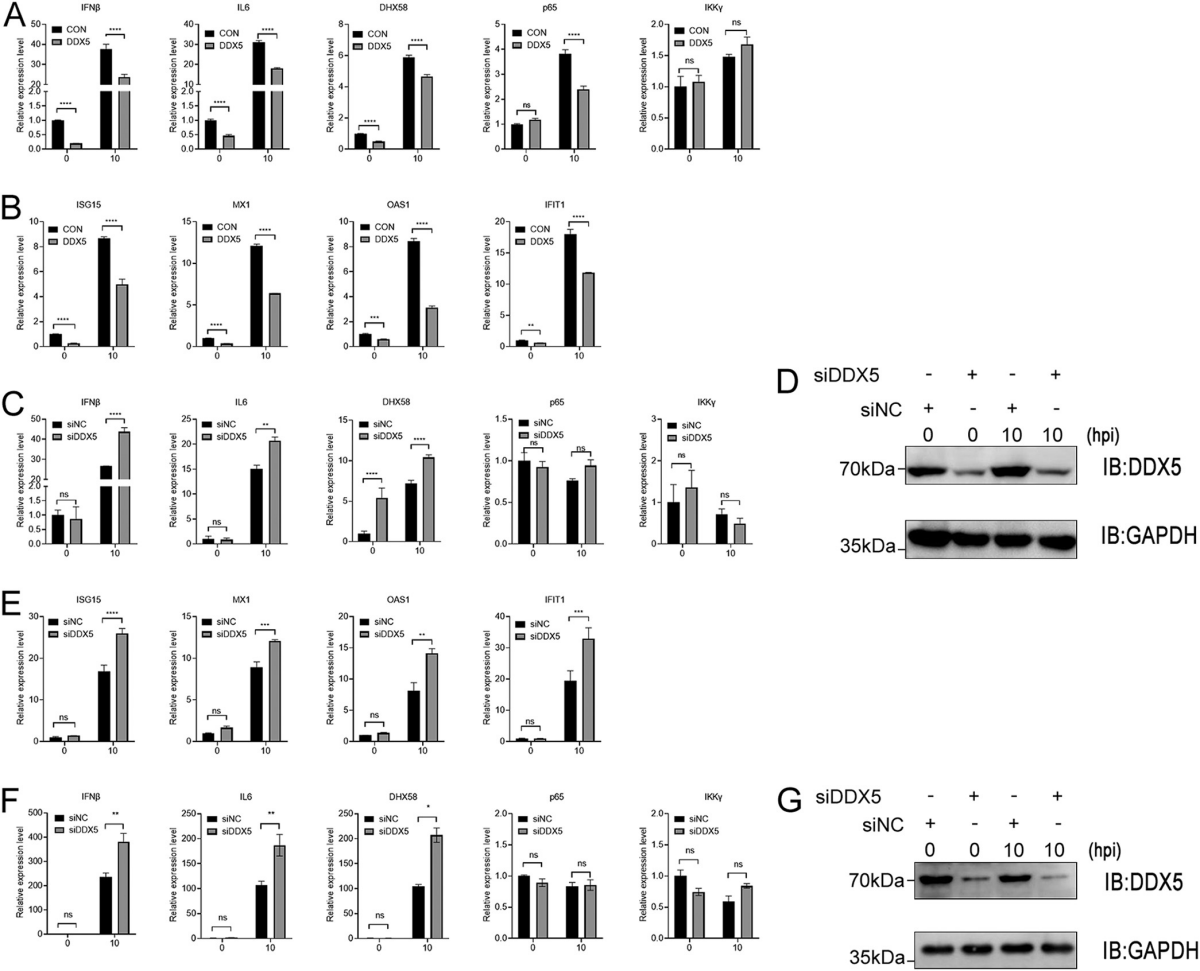
DDX5 suppresses IAV-triggered IFN-β and ISG production. (A and B) A549 cells were transfected with MYC-DDX5 or empty pCAGGS vector. At 24 h after transfection, cells were infected with WSN/1933 (MOI = 0.5), and they were harvested at 0 h and 10 h after infection. The mRNA levels of IFN-β, IL-6, DHX58, p65, and IKKγ (A) and of ISG15, MX1, OAS1, and IFIT1 (B) were assessed by qPCR. (C, D, and E) A549 cells were transfected with siRNA targeting DDX5 or with scrambled siRNA for 48 h. The cells were infected with WSN/1933 (MOI = 0.5) and harvested at 0 h and 10 h after infection. The mRNA levels of IFN-β, IL-6, DHX58, p65, and IKKγ (C) and of ISG15, MX1, OAS1, and IFIT1 (E) were assessed by qPCR. (D) The knockdown effect of DDX5 was detected by Western blotting. (F and G) A549 cells were transfected with siRNA targeting DDX5 or with scrambled siRNA for 48 h. The cells were infected with WSN-H7 (MOI = 1) and harvested at 0 h and 10 h after infection. (F) The mRNA levels of IFN-β, IL-6, DHX58, p65, and IKKγ were assessed by qPCR. (G) The knockdown effect of DDX5 was detected by Western blotting. Experiments were independently repeated three times, with similar results. The results are presented as mean values ± standard deviations. *, *P* < 0.05; **, *P* < 0.01; ***, *P* < 0.001; ****, *P* < 0.0001; ns, no significance.

By sensing viral nucleic acids, host innate receptors elicit signaling pathways converging on the activation of protein kinase TBK1, as well as mitogen-activated protein (MAP) kinases (MAPKs), which in turn activate the transcription factor IRF3 and NF-κB to induce IFN-I and proinflammatory cytokine production, respectively (28). Mouse DDX5 promotes the m6A modification and nuclear export of transcripts of DHX58, p65, and IKKγ in mouse embryo fibroblasts (MEFs), and DDX5 serves as a negative regulator of innate immunity (34). We found that the homology between human and mouse DDX5 proteins was very high, with only a few amino acids differing at the C terminus of the protein and one amino acid less in the human DDX5 than in the mouse DDX5 (Fig. S2). To further investigate whether human DDX5 negatively regulates viral infection-induced IFN responses, we then detected the IFN-I signaling in A549 cells infected with WSN/1933. We found that DDX5 overexpression suppressed IAV infection-induced upregulation of IFN-β, IL-6, DHX58, and p65 but not IKKγ (Fig. 5A).

To further analyze the role of DDX5 in IFN-β-mediated signaling, we examined IFN-β-induced downstream antiviral genes. Quantitative real-time PCR analysis also indicated that transcripts of ISG15, MX1, OAS1, and IFIT1 were downregulated in DDX5-overexpressing A549 cells infected with WSN/1933 virus (Fig. 5B). Conversely, knocking down DDX5 led to elevated transcripts of IFN-β, IL-6, and DHX58 but not p65 and IKKγ (Fig. 5C). Similarly, silencing DDX5 promoted transcription of antiviral genes downstream from IFN-β, including genes encoding ISG15, MX1, OAS1, and IFIT1 (Fig. 5E). Of course, we also examined the knockdown of DDX5 in the above-described experiments (Fig. 5D). Knockdown of DDX5 also upregulated the antiviral transcripts of IFN-β, IL-6, and DHX58 in A549 cells infected with recombinant influenza virus WSN-H7 at high doses (Fig. 5F and G). To summarize, human DDX5 downregulated the expression of IFN-β and IL-6 and the transcription of antiviral genes downstream from IFN-β in influenza virus-infected A549 cells.

### METTL3-METTL14/YTHDF2 axis promotes RNA decay of antiviral transcripts.

**FIG 6:**
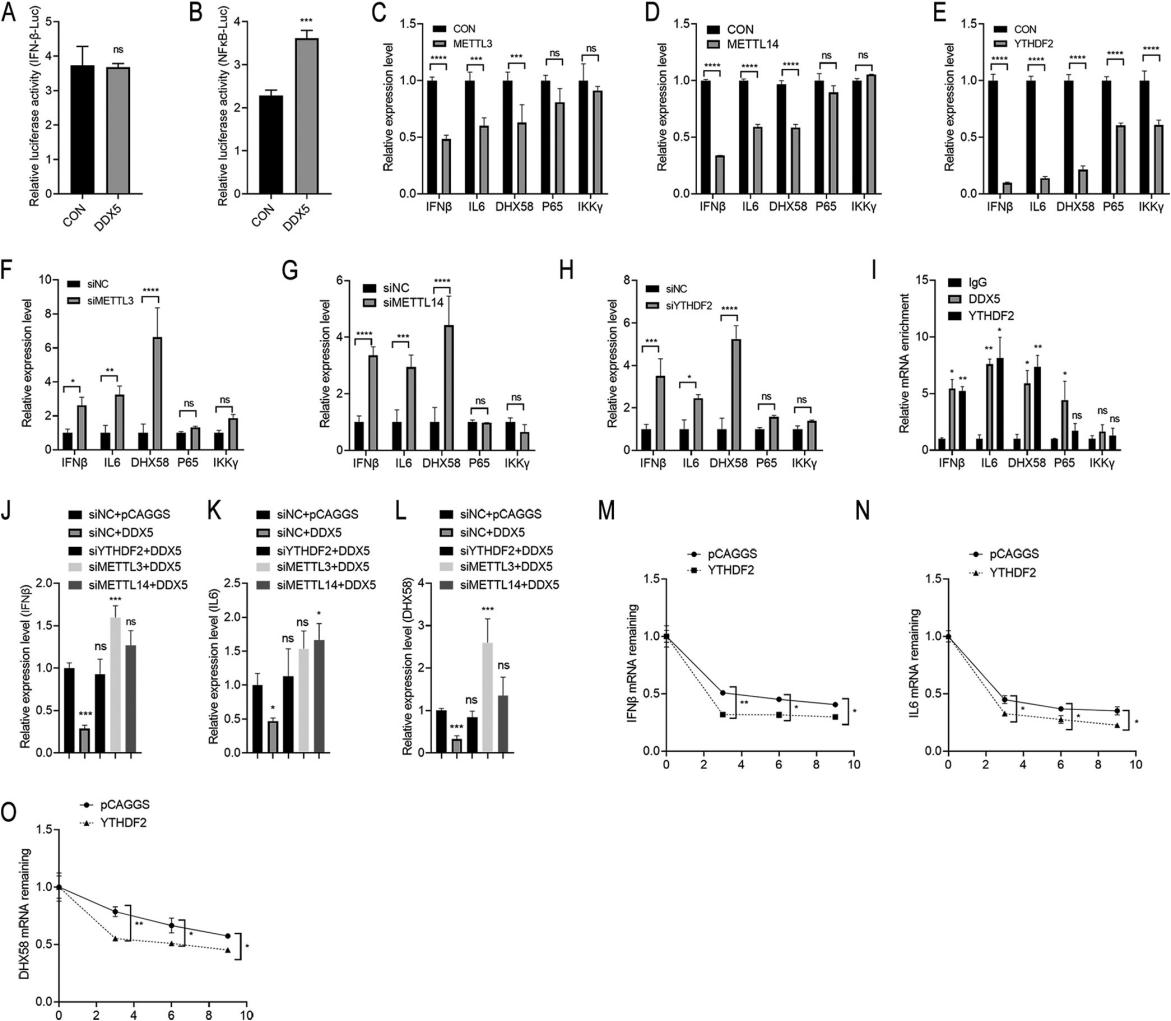
The METTL3-METTL14/YTHDF2 axis promotes RNA decay of antiviral transcripts. (A and B) IFN-β–Luc (A) or NF-κB–Luc (B) reporter plasmid and plasmid pGL4.75 together with DDX5 or empty vector were cotransfected into HEK293T cells, and then cells were infected with SeV at 24 h after transfection. Cells were lysed at 24 h after infection. Firefly luciferase and *Renilla* luciferase activities were determined with the dual-luciferase reporter assay system (Promega). (C, D, and E) A549 cells were transfected with METTL3 (C), METTL14 (D), YTHDF2 (E) expression plasmid or empty pCAGGS vector. At 24 h after transfection, cells were harvested. The mRNA levels of IFN-β, IL-6, DHX58, p65, and IKKγ were assessed by qPCR. (F, G, and H) A549 cells were transfected with siRNA targeting METTL3 (F), METTL14 (G), or YTHDF2 (H) or with scrambled siRNA for 48 h. Cells were harvested, and the mRNA levels of IFN-β, IL-6, DHX58, p65, and IKKγ were assessed by qPCR. (I) A549 cells were infected with WSN/1933 (MOI = 0.5) for 12 h and subjected to DDX5 and YTHDF2 RIP-qPCR to detect IFN-β, IL-6, DHX58, p65, and IKKγ. The results are presented relative to those obtained with control groups. (J, K, and L) A549 cells were transfected with siRNA targeting YTHDF2, METTL3, or METTL14 or with scrambled siRNA for 24 h. Then, the cells were transfected with MYC-DDX5 expression plasmid or empty pCAGGS vector. At 48 h after transfection, cells were harvested and the mRNA levels of IFN-β (J), IL-6 (K), and DHX58 (L) were assessed by qPCR. (M, N, and O) A549 cells were transfected with YTHDF2 expression plasmid or empty pCAGGS vector. At 24 h after transfection, cells were infected with WSN/1933 (MOI = 0.5). Cells were treated with actinomycin D at 12 h after infection and harvested at 0 h, 3 h, 6 h, and 9 h. The mRNA levels of IFN-β (M), IL-6 (N), and DHX58 (O) were assessed by qPCR. Residual RNAs were normalized to 0 h. Experiments were independently repeated three times, with similar results. The results are presented as mean values ± standard deviations. *, *P* < 0.05; **, *P* < 0.01; ***, *P* < 0.001; ****, *P* < 0.0001; ns, no significance.

To further investigate the role of DDX5 in the IFN-β signaling pathway, we investigated the promoter activity of IFN-β and NF-κB in DDX5-overexpressing HEK293T cells. Luciferase reporter assays showed that DDX5 had no significant effect on IFN-β promoter activity (Fig. 6A) but slightly promoted NF-κB promoter activity (Fig. 6B), indicating that DDX5 negatively regulates IFN-β and IL-6 production in other ways. It was reported that DDX5 interacted with the *N*^6^-methyladenosine (m6A) writer METTL3 to regulate methylation of mRNA through affecting the m6A writer METTL3-METTL14 heterodimer complex (34). Additionally, mRNAs bound specifically to DDX5 at the conserved GCUGCAG motif. Surprisingly, human IFN-β, IL-6, DHX58, p65, and IKKγ mRNA transcripts all contain one or more GCUGCAG motifs. However, only murine DHX58 and IKKγ mRNA transcripts contained the conserved motifs, while murine IFN-β, IL-6, and p65 mRNA transcripts did not (Table S3). The above-described phenomenon led us to speculate that the DDX5/METTL3-METTL14 axis regulated antiviral transcripts, thereby participating in immune regulation. Then, we constructed METTL3 and METTL14 protein expression plasmids. We found that overexpression of METTL3 or METTL14 in A549 cells significantly downregulated the mRNA levels of IFN-β, IL-6, and DHX58 but had no effect on the mRNA levels of p65 and IKKγ, which was consistent with the regulatory mode of DDX5 (Fig. 6C and D).

RNA decay has been reported to play roles in several biological processes of mRNA m6A methylation, and YTHDF2, a key reader protein for m6A methylation of RNA transcripts, promotes cytoplasmic mRNA degradation (46, 47). The results showed that overexpression of YTHDF2 significantly downregulated the above-named transcripts, including IFN-β, IL-6, DHX58, p65, and IKKγ (Fig. 6E). From the above-described results, we could speculate that the METTL3-METTL14/YTHDF2 axis regulated the mRNA levels of IFN-β, IL-6, and DHX58. To further confirm our hypothesis, we knocked down METTL3, METTL14, or YTHDF2 by siRNA-mediated silencing. The results showed that silencing METTL3 (Fig. 6F), METTL14 (Fig. 6G), or YTHDF2 (Fig. 6H) significantly upregulated the mRNA levels of IFN-β, IL-6, and DHX58 in A549 cells. At the same time, we also detected the corresponding silencing effects of METTL3, METTL14, and YTHDF2 in A549 cells (Fig. S3). To confirm the association between DDX5 and METTL3-METTL14/YTHDF2-mediated regulation of the transcripts, we analyzed the abundances of IFN-β, IL-6, DHX58, p65, and IKKγ transcripts via RNA immunoprecipitation-quantitative PCR (RIP-qPCR). We found that mRNA transcripts of IFN-β, IL-6, and DHX58 were significantly enriched on DDX5- and YTHDF2-coupled agarose beads, while there was no significant change in the abundance of p65 and IKKγ transcripts (Fig. 6I). We further found that silencing METTL3, METTL14, or YTHDF2 blocked the downregulation of the mRNA levels of IFN-β (Fig. 6J), IL-6 (Fig. 6K), and DHX58 (Fig. 6L) by overexpressing DDX5 in A549 cells, which implied that DDX5 regulated the above-named transcripts via the METTL3-METTL14/YTHDF2 axis. It is reported that m6A-methylated mRNA in the cytoplasm binds to cytosolic reader proteins that affect the stability, translation, or localization of mRNAs (39, 48). Next, we aimed to determine the stability of the above-mentioned transcripts, IFN-β, IL-6, and DHX58, using an RNA decay assay. The stability of IFN-β, IL-6, and DHX58 transcripts (Fig. 6M to O) when YTHDF2 was overexpressed in A549 cells was significantly decreased after RNA synthesis was inhibited using actinomycin D. In summary, these results indicated that the DDX5/METTL3-METTL14/YTHDF2 axis regulated the mRNA levels of antiviral transcripts of IFN-β, IL-6, and DHX58.

### METTL3-METTL14/YTHDF2 axis positively regulates the replication of influenza virus.

**FIG 7:**
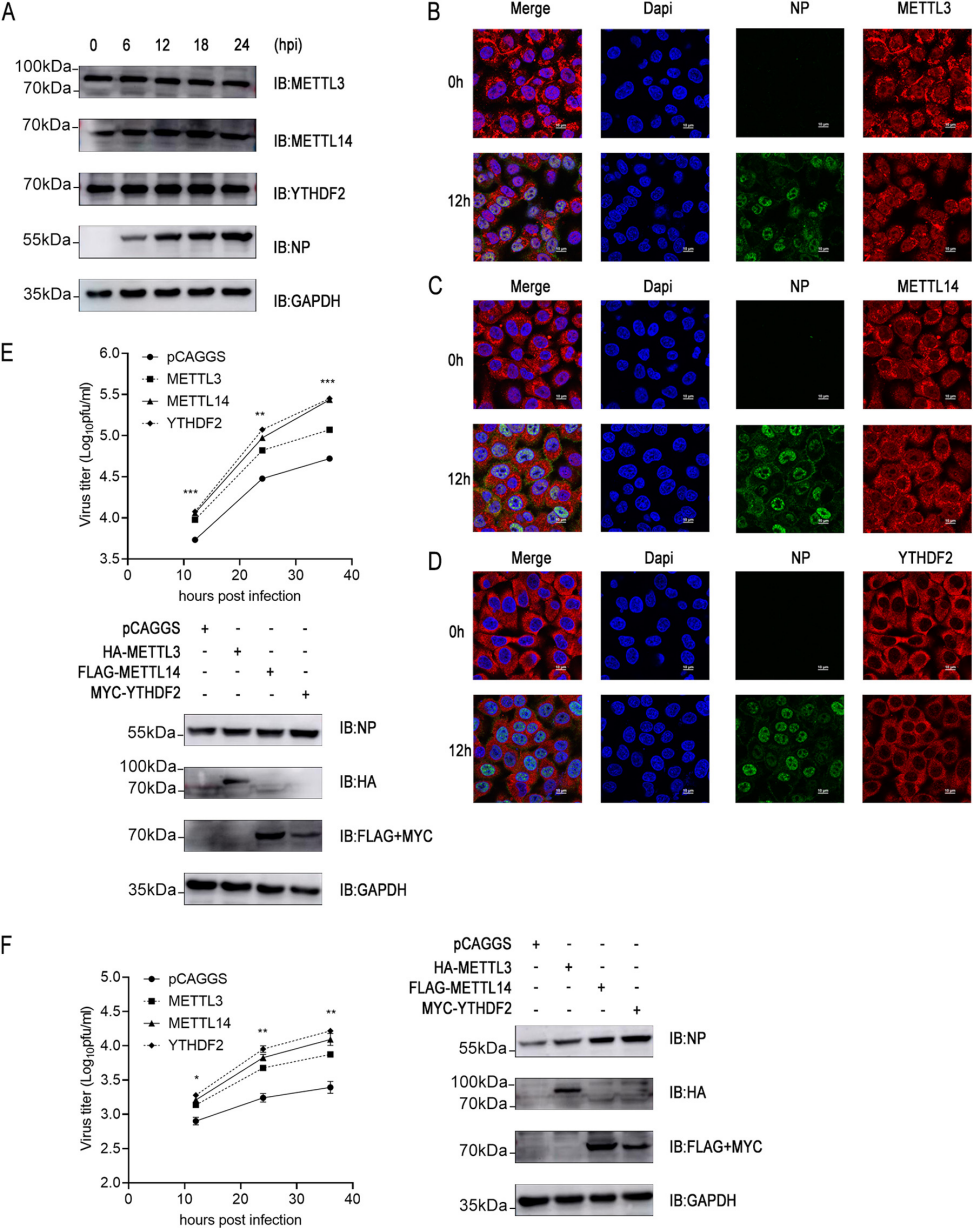
METTL3-METTL14/YTHDF2 axis positively regulates the replication of influenza virus. (A) A549 cells were infected with influenza virus WSN/1933 at an MOI of 0.5. At 0 h, 6 h, 12 h, 18 h, and 24 h after infection, the cells were lysed to harvest the protein lysate, and protein expression levels were detected by Western blotting. (B, C, and D) A549 cells were infected with influenza virus WSN/1933 at an MOI of 0.5. At 0 h and 12 h after infection, the cells were fixed, permeabilized, and stained with anti-METTL3 antibody (B), anti-METTL14 antibody (C), anti-YTHDF2 antibody (D), and anti-NP antibody, followed by immunostaining with A546-labeled goat anti-mouse secondary antibody or FITC-labeled goat anti-rabbit secondary antibody. Then, cells were stained with DAPI and examined via fluorescence microscopy. (E and F) A549 cells were transfected with HA-METTL3, FLAG-METTL14, or MYC-YTHDF2 expression plasmid or empty pCAGGS vector. At 24 h after transfection, cells were infected with WSN-H7 (MOI = 0.01) (E) or WSN/1933 (MOI = 0.01) (F). The supernatants were sampled at 12 h, 24 h, and 36 h after infection, and viral titers in the supernatants were determined by plaque assay. The expression levels of HA-METTL3, FLAG-METTL14, MYC-YTHDF2, and viral NP were measured by Western blotting at 24 h after infection. Experiments were independently repeated three times, with similar results. The results are presented as mean values ± standard deviations. Statistical differences between the METTL3 overexpression group and the control group (pCAGGS) according to two-way ANOVA are indicated as follows: *, *P* < 0.05; **, *P* < 0.01; ***, *P* < 0.001.

The m6A machinery is required for human cytomegalovirus (CMV) propagation (49, 50). However, the role of the METTL3-METTL14/YTHDF2 axis during influenza virus replication has not been reported. First, we examined the protein expression of the METTL3-METTL14/YTHDF2 axis in influenza virus-infected A549 cells. The results showed that the protein levels of METTL3, METTL14, and YTHDF2 did not change significantly in A549 cells during infection by influenza virus WSN/1933 (Fig. 7A). Then, we further observed the subcellular distribution of METTL3, METTL14, and YTHDF2 during influenza virus infection. METTL3, METTL14, and YTHDF2 showed different nucleocytoplasmic distributions. YTHDF2 was mainly distributed in the cytoplasm, which was consistent with its function. However, METTL3 was mainly localized in the nucleus, with a small distribution in the cytoplasm. METTL14 was abundantly distributed in the nucleus and cytoplasm. Surprisingly, influenza virus infection did not significantly affect the nucleocytoplasmic distribution of METTL3, METTL14, and YTHDF2 (Fig. 7B to D).

To further illustrate the roles of METTL3, METTL14, and YTHDF2 in influenza virus replication, A549 cells transfected with METTL3, METTL14, or YTHDF2 expression plasmids were infected with influenza virus. The results showed that overexpression of METTL3, METTL14, or YTHDF2 significantly promoted the viral replication of influenza viruses WSN-H7 and WSN/1933 (Fig. 7E and F). As expected, overexpression of METTL3, METTL14, or YTHDF2 also promoted the accumulation of viral proteins (Fig. 7E and F). Taken together, the METTL3-METTL14/YTHDF2 axis promotes the replication of influenza virus.

## DISCUSSION

In this study, we elucidated DDX5, a prototypical member of the large ATP-dependent RNA helicase family, as a proviral factor involved in influenza virus replication. DEAD/H-box RNA helicases are inextricably linked to influenza viruses. DDX1, DDX56, DDX39B, DDX3X, DDX19, DDX17, DDX21, DHX9, and DDX58 have all been reported to be involved in influenza virus replication (Table S2), with some of them positively regulating viral replication and, conversely, some negatively regulating viral replication. It has been established that DEAD/H-box RNA helicases participate in cell cycle regulation, tumorigenesis, apoptosis, cancer development, adipogenesis, Wnt–β-catenin signaling, and viral infection (9). Whether other members of the DEAD/H-box RNA helicases are involved in influenza virus replication is a particularly intriguing question. We confirmed previous conclusions and also found novel DEAD/H-box RNA helicases involved in influenza virus replication (Fig. 1C and D). Interestingly, many DEAD/H-box RNA helicases play multiple roles in the viral replication cycle, some of which even seem contradictory. RNA helicase DDX3 is at the crossroads of viral replication and antiviral immunity, being a component of the innate immune response against viral infection and hijacked to accomplish steps of viral replication (51). Recently, it has been reported, and our laboratory also found, that DDX39B and DDX39A are involved in the nucleocytoplasmic transport of influenza virus mRNA, being able to combine with some antiviral transcripts to restrict their expression (52), and also participate in the regulation of viral polymerase activity (our data are not yet published). Of course, DDX5 is no exception, being important for the viral replication of severe acute respiratory syndrome coronavirus (SARS-CoV) (53), hepatitis C virus (HCV) (54), and porcine reproductive and respiratory syndrome virus (PRRSV) (55). However, DDX5 inhibits viral replication for two DNA viruses, hepatitis B virus (HBV) and myxoma virus (MYXV) (56, 57). Another interesting phenomenon is that many DEAD/H-box RNA helicases bind to NP or NS1 of influenza virus. In our study, we found that DDX5 interacted with NP of influenza virus, independent of RNA. Additionally, DDX5 interacted with the full length of NP through the N terminus (Fig. 3). Then, based on the interaction of DDX5 and NP, we speculated that influenza virus infection may cause changes in the expression and nucleocytoplasmic distribution of DDX5, such as have been reported in other viral infections. Surprisingly, infections with multiple strains of influenza virus had no effect on the protein expression level and cellular distribution of DDX5 (Fig. 4). The biological significance of the DDX5-NP interaction needs to be further elucidated in future studies.

Furthermore, DDX5 could inhibit IFN-β and IL-6 production after infection with influenza virus, which suggests that DDX5 may be involved in regulating antiviral innate signaling (Fig. 5). Our findings are also in line with those of a recent study that reported that DDX5 interacted with METTL3 in the nucleus of VSV (vesicular stomatitis virus)-infected MEFs and regulated DHX58 and NF-κB transcripts (34). Interestingly, human mRNA transcripts of IFN-β, IL-6, and DHX58 contained many DDX5-binding motifs but murine transcripts did not. Consistently, the mRNA transcripts of murine DHX58 and IKKγ contained the above-mentioned motifs (Table S3). Species differences and strain characteristics in the regulation of viral replication by DDX5 have to be considered. The mechanism by which DDX5 regulates influenza virus replication in A549 cells is obscure and needs to be elucidated. Most of the DEAD/H-box RNA helicases are conserved, but DHX9 and RIG-I are genetically deficient in chickens, which may cause exacerbation of viral infections like influenza in chickens. Therefore, we hypothesized that human DDX5 may operate in regulating transcription to manipulate the innate immune response in A549 cells. As we expected, overexpression of DDX5 downregulated the mRNA levels of IFN-β, IL-6, and DHX58 and the transcripts of ISGs. Silencing DDX5 has the opposite results (Fig. 5). Then, we further ruled out the possibility that DDX5 played a role in the production upstream from IFN-β by performing IFN-β and NF-κB promoter activity reporter experiments. Based on the above-described experimental results, it was not difficult to speculate that DDX5 may regulate immune response-related transcripts at the posttranscriptional level through the METTL3-METTL14 complex. We found that overexpression of METTL3 or METTL14 significantly downregulated the mRNA levels of IFN-β, IL-6, and DHX58. Next, overexpression of YTHDF2 significantly downregulated the mRNA levels of IFN-β, IL-6, and DHX58, suggesting that these transcripts are regulated by the METTL3-METTL14/YTHDF2 axis. Silencing of METTL3, METTL14, or YTHDF2 significantly upregulated the mRNA levels of IFN-β, IL-6, and DHX58, which further demonstrated the above-mentioned regulatory axis. We further assumed that DDX5 exerted responsibility for the RNA degradation of antiviral transcripts via the METTL3-METTL14/YTHDF2 axis. Indeed, DEAD/H-box RNA helicases, such as DDX46, have been reported to be involved in the regulation of antiviral transcripts by affecting the m6A modification process (23). To confirm our hypothesis, we analyzed the binding of YTHDF2 or DDX5 and transcripts and found that the mRNAs of IFN-β, IL-6, and DHX58 were significantly enriched. Next, we demonstrated through rescue experiments that DDX5 regulates transcripts dependently on the METTL3-METTL14/YTHDF2 axis. The results showed that overexpression of DDX5 no longer downregulated the mRNA levels of IFN-β, IL-6, and DHX58 when METTL3, METTL14, or YTHDF2 protein was knocked down in A549 cells. Finally, we found that overexpression of YTHDF2 accelerated the mRNA decay of IFN-β, IL-6, and DHX58 (Fig. 6). The above-described results confirmed that the DDX5/METTL3-METTL14/YTHDF2 axis regulated the transcript levels of IFN-β, IL-6, and DHX58. However, the levels and positions of m6A modifications in the above-named antiviral transcripts need to be further explored. Whether DDX5 regulates other transcripts in addition to the above-mentioned antiviral transcripts is a question worthy of further study. A previous study showed that m6A modification controls the innate immune response to infection (49). We further analyzed the role of the METTL3-METTL14/YTHDF2 axis in influenza virus replication. The results showed that this signaling axis positively regulated viral replication, which was consistent with the experimental results showing that the METTL3-METTL14/YTHDF2 axis negatively regulated antiviral transcripts like those of IFN-β and IL-6 (Fig. 7).

In summary, our findings provided a novel perspective to understand the mechanism by which DDX5 regulates antiviral immunity. In addition, our results reveal new insight into the roles of DExD/H-box protein family members in influenza virus replication.

## MATERIALS AND METHODS

### Viruses and cells.

Human embryonic kidney cells (HEK293T cell line) were cultured in Dulbecco’s modified Eagle’s medium (DMEM; Invitrogen) containing 10% fetal bovine serum (FBS; Gibco), 0.2% NaHCO_3_, 100 μg/mL streptomycin, and 100 IU/mL penicillin (Gibco) at 37°C with 5% CO_2_. Madin-Darby canine kidney (MDCK) cells were grown in DMEM containing 10% fetal bovine serum (FBS; Sigma). A549 cells were grown in DMEM containing 10% FBS (Sigma). Influenza A viruses A/WSN/1933 (H1N1) and CK/SH/49/19 (H9N2) and reassortant virus WSN-H7 (H1N1) were generated by reverse genetics and inoculated into 10-day-old specific-pathogen-free (SPF) chicken embryos for virus propagation. For biosafety reasons, we rescued the reassortant virus WSN-H7 (H1N1) using polymerase (PB2, PB1, and PA subunits) and nucleoprotein (NP) genes from A/Anhui/1/2013 (H7N9) and the remaining viral genes (hemagglutinin [HA], neuraminidase [NA], matrix protein [M], and nonstructural protein [NS]) from the laboratory-adapted A/WSN/1933 (H1N1) influenza virus strain. Viral titers were measured by plaque assay in MDCK cells.

### Plasmid construction.

Cellular RNA from A549 cells was extracted by lysing the cells with TRIzol reagent (R0016; Beyotime). The RNA was reverse transcribed into single-stranded DNA using the RevertAid first-strand cDNA synthesis kit (K1622; Thermo Fisher). Full-length DNA fragments encoding DDX3X, DDX5, DHX9, DDX15, DDX17, DDX58, DDX27, METTL3, METTL14, or YTHDF2 were amplified by PCR, and the PCR products were inserted into the vector pCAGGS. To facilitate Western blotting detection, an HA tag, MYC tag, or Flag tag was added to the indicated gene fragments. All plasmid constructs were verified by Sanger sequencing. All primers and sequences used in this article are available from the authors upon request.

### Western blotting and antibodies.

Cells were washed three times with cold phosphate-buffered saline (PBS) and lysed in cold lysis buffer (1% Triton X-100, 1 mM phenylmethylsulfonyl difluoride [PMSF] in PBS) for 30 min. Lysates were clarified by centrifugation at 12,000 × *g* for 10 min. Proteins in the lysates were separated by SDS-PAGE, transferred to nitrocellulose membranes (catalog number 10600001; GE Amersham), and then probed with the antibodies indicated in the figure legends. Finally, the membranes were incubated with enhanced chemiluminescence (ECL) reagents (Vazyme, China), and the signals were analyzed using an Amersham Imager 600 charge-coupled (device) CCD-based chemiluminescent analyzer (GE Healthcare). Proteins were detected with the following antibodies: anti-glyceraldehyde-3-phosphate dehydrogenase (GAPDH) antibody (10494-1-AP; Proteintech), anti-DDX5 antibody (10804-1-AP; Proteintech), anti-METTL3 antibody (15073-1-AP; Proteintech), anti-METTL14 antibody (26158-1-AP; Proteintech), anti-YTHDF2 antibody (24744-1-AP; Proteintech), anti-lamin B1 antibody (66095-1-Ig; Proteintech), anti-Flag monoclonal antibody (MAb) (F1804; Sigma-Aldrich), anti-MYC MAb (60003-2-Ig; Proteintech), and anti-HA MAb (H9658; Sigma-Aldrich). In addition, anti-NP antibodies were kindly provided by Chengjun Li (Harbin Veterinary Research Institute, the Chinese Academy of Agricultural Sciences).

### Coimmunoprecipitation (co-IP).

Transiently transfected cells were washed twice with PBS and lysed in NP-40 lysis buffer (product number P0013F; Beyotime) supplemented with protease inhibitor (P1005; Beyotime). Whole-cell lysate was first precleared with protein A/G slurry (catalog number sc-2003; Santa Cruz). After centrifugation at 1,000 × *g* for 5 min at 4°C, supernatant along with 1 μg of mouse anti-Flag MAb and 50 μL of protein A/G slurry (catalog number sc-2003; Santa Cruz) was incubated with rotation for 4 h at 4°C. Immunoprecipitated samples collected by centrifugation were washed with NP-40 lysis buffer four times. The final pellet was dissolved in 4× SDS loading buffer (catalog number P1016; Solarbio) for SDS-PAGE and Western blotting.

### Isolation of nuclear and cytoplasmic protein.

Nuclear and cytoplasmic protein were isolated separately using the nuclear and cytoplasmic protein extraction kit (catalog number P0027; Beyotime) according to the manufacturer’s protocol.

### RNA immunoprecipitation (RIP).

A549 cells were grown in a 6-well plate, and the cells were infected with WSN (multiplicity of infection [MOI] of 1) for 12 h. Then, the infected cells were harvested and lysed for immunoprecipitation with anti-DDX5, anti-YTHDF2 or anti-rabbit IgG antibody. Immunoprecipitated RNA was extracted from immunoprecipitated samples, and cDNA was synthesized with oligo(dT)_20_ for mRNA. The relative enrichment of each transcript was determined through quantitative real-time PCR. Relative enrichment was first normalized to the input and then analyzed by comparison with the data from the immunoprecipitated sample.

### RNA decay assays.

To measure the stability of RNA transcripts, we performed the RNA decay assay in A549 cells by adding actinomycin D at a final concentration of 5 μM. The assay was performed according to the method described in a previous study (49).

### Viral polymerase-minigenome assay.

To evaluate the effects of various cellular factors on influenza virus polymerase activity, HEK293T cells were transfected in 24-well plates with pCAGGS plasmids encoding the PB2 (100 ng), PB1 (100 ng), PA (100 ng), and NP (100 ng) proteins, together with 50 ng species-specific minigenome reporter plasmids (PolI-Luc), 50 ng *Renilla* luciferase expression plasmids (RL-TK) as an internal control, and plasmids (250 ng) encoding various cellular factors, using ExFect transfection reagent (Vazyme) according to the manufacturer’s instructions. Cells were incubated at 37°C. At 48 h after transfection, cells were lysed with 100 μL of passive lysis buffer (Promega), and firefly and *Renilla* luciferase bioluminescence was measured using a dual-luciferase system (Promega). The expression levels of polymerase subunit PB2, NP, or cellular factors in different groups were assessed by Western blotting using specific antibodies.

### Luciferase reporter assay.

HEK293T cells grown in 24-well plates were cotransfected with 0.25 μg/well of IFN-β–Luc or NF-κB–Luc reporter plasmid, 0.01 μg/well of plasmid pGL4.75 (hRluc/CMV), 0.25 μg/well DDX5 protein expression plasmid, or empty vector for 24 h. Then, cells were infected with Sendai virus (SeV) at 24 h posttransfection (h.p.i.). Cells were lysed at 24 h.p.i., and firefly luciferase and *Renilla* luciferase activities were determined with the dual-luciferase reporter assay system (Promega) according to the manufacturer’s protocol. Data are presented as relative firefly luciferase activities normalized to *Renilla* luciferase activities and are representative of three independent experiments.

### siRNA-mediated silencing.

A549 cells were transfected with 20 nM target gene siRNA or the negative control (NC.si) using Lipofectamine 2000 (Invitrogen) in 24-well plates, according to the manufacturer’s instructions. Twenty-four hours later, cells were infected with influenza virus. siRNAs and the negative control (NC.si) were purchased from GenePharma (Shanghai, China). siRNAs for target genes were as follows: siDDX5, 5′-ACAUAAAGCAAGUGAGCGA-3′; siMETTL3, 5′-GCCAAGGAACAAUCCAUUGUU-3′; siMETTL14, 5′-CCAUGUACUUACAAGCCGAUA-3′; siYTHDF2, 5′-UCUGGAUAUAGUAGCAAUUAU-3′; and NC.si: 5′-UUCUCCGAACGUGUCACGUTT-3′.

### qPCR.

Influenza virus-infected cells were washed three times with PBS, and total RNA was extracted using TRIzol reagent (catalog number R0016; Beyotime). Total RNA (1,000 ng) was subjected to first-strand cDNA synthesis with oligo(dT)_20_ using the HiScript II 1st-strand cDNA synthesis kit (Vazyme, China). Quantitative real-time PCR was then performed using the cDNAs, gene-specific primer pairs, and AceQ qPCR SYBR green master mix (Vazyme, China) in a Roche LightCycler 96 according to the manufacturer’s instructions, using the following cycling program: 95°C for 5 min and 40 cycles of 95°C for 10 s and 60°C for 30 s. The cycle threshold (2^−ΔΔ^*^CT^*) method was used to determine the relative levels of candidate genes. Gene-specific primer pairs were as follows: IFN-β, 5′-AGGACAGGATGAACTTTGAC-3′ and 5′-TGATAGACATTAGCCAGGAG-3′; IL-6, 5′-AGAGGCACTGGCAGAAAACAAC-3′ and 5′-AGGCAAGTCTCCTCATTGAATCC-3′; DHX58, 5′-GTGCATCATGTCACCCCAGA-3′ and 5′-AGCTTCTTCAGCAAGTCCCC-3′; P65, 5′-CTATAGAAGAGCAGCGTGGGG-3′ and 5′-TCACTCGGCAGATCTTGAGC-3′; IKKγ, 5′-GCCAGGATCGAGGACATGAG-3′ and 5′-ATACTGGCACTTGGGACAGC-3′; and GAPDH, 5′-GCCAAGGCTGTGGGCAAGG-3′ and 5′-GGAGGAGTGGGTGTCGCTG-3′.

### Immunofluorescence.

A549 or HEK293T cells were seeded on glass coverslips and transfected with indicated plasmids or infected with indicated influenza viruses. After incubation, cells were fixed with 4% paraformaldehyde and permeabilized with a solution of PBS containing 0.2% Triton X-100 for 10 min (Sigma). Then, the cells were washed and stained for 1 h with primary antibodies, followed by staining with fluorescent secondary antibodies in 5% bovine serum albumin (BSA; Thermo Scientific) solution in PBS. Antibodies used in this study were mouse anti-NP MAb, goat anti-rabbit Alexa Fluor 546-labeled secondary antibody (catalog numberA21085; Invitrogen), and goat anti-mouse fluorescein isothiocyanate (FITC)-labeled secondary antibody (172-1806; KPL). After staining with secondary antibodies, cells were stained with 4,6-diamidino-2-phenylindole (DAPI) for 10 min. Finally, images were acquired using a confocal microscope (Nikon, Japan) equipped with a Plan Apo objective (60×/1.40, oil immersion; Nikon, Japan) and CFI eyepiece (10×/22; Nikon) (58).

### Plaque assays.

The infectious titers of influenza viruses were determined by plaque assays. Briefly, viruses were serially 10-fold diluted and inoculated onto MDCK cell monolayers. After incubation at 37°C for 60 min, the cells were overlaid with DMEM containing 1% SeaPlaque agarose (Lonza) and 1 μg/mL TPCK (tosylsulfonyl phenylalanyl chloromethyl ketone)-trypsin and incubated at 37°C. At 2 days after infection, cultures were checked for plaque formation.

### Phylogenetic trees of DEAD/H-box helicases.

The evolutionary history was inferred using the neighbor-joining method. The percentages of replicate trees in which the associated taxa clustered together in the bootstrap test (500 replicates) are shown next to the branches in the figure. Evolutionary analyses were conducted in MEGA6.

### Statistical analysis.

The data are shown as mean values ± standard deviations, and unless otherwise indicated, all the data presented are representative results of at least three independent repeats. Statistical analysis was performed with Prism 8 (GraphPad), and the statistics were analyzed by two-tailed Student’s *t* test or one-way or two-way analysis of variance (ANOVA) as indicated. Differences considered to be significant are indicated by asterisks as follows: *, *P* < 0.05; **, *P* < 0.01; ***, *P* < 0.001; ****, *P* < 0.0001. “ns” indicates no significance.
