# Supplementary material for: DDX5/METTL3-METTL14/YTHDF2 Axis Regulates Replication of Influenza A Virus
Source: Microbiol Spectr. 2022 May 18;10(3):e01098-22. doi: 10.1128/spectrum.01098-22 (PMC9241928; doi:10.1128/spectrum.01098-22)
Supplement: SUPPLEMENTAL FILE 1 — Supplemental material. Download spectrum.01098-22-s001.pdf, PDF file, 0.3 MB [file spectrum.01098-22-s001.pdf]

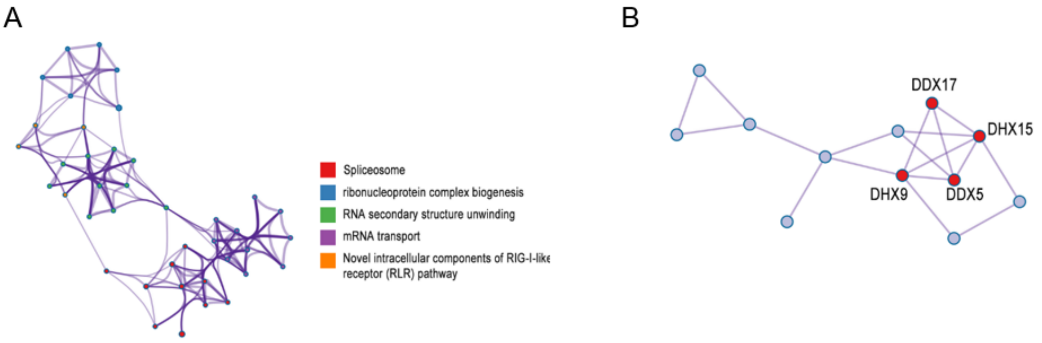

FIGS1 Related analysis of multiple DExH-box helicases. (A) Analysis was performed using Metascape (http://metascape.org). To further capture the relationships between the terms, a subset of enriched terms were selected and rendered as a network plot. (B) Protein-protein interaction network and MCODE components were identified.

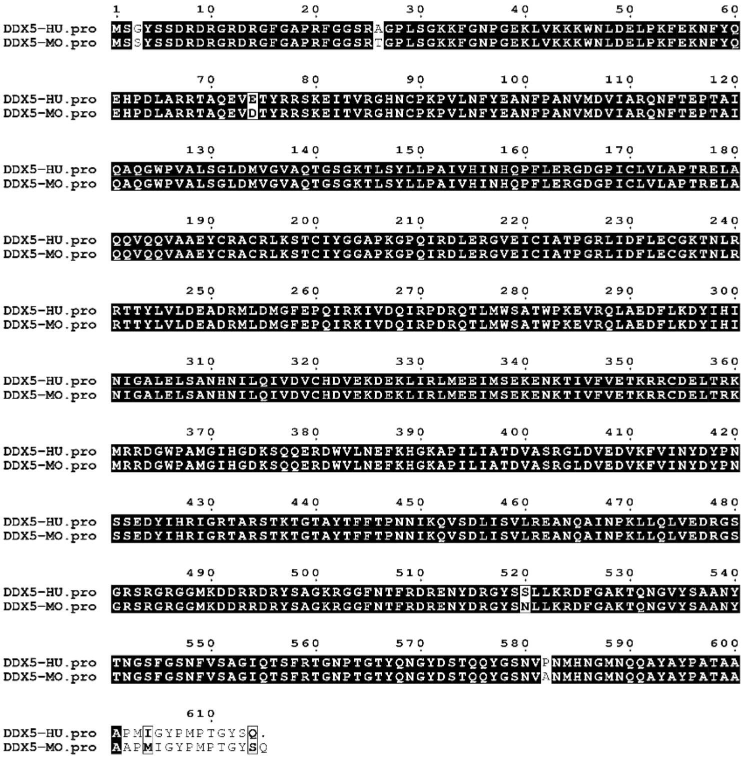

FIGS2 Alignment of amino acids of DDX5 proteins. All sequences were aligned with ClustalW and mapped online program, ESPript server.

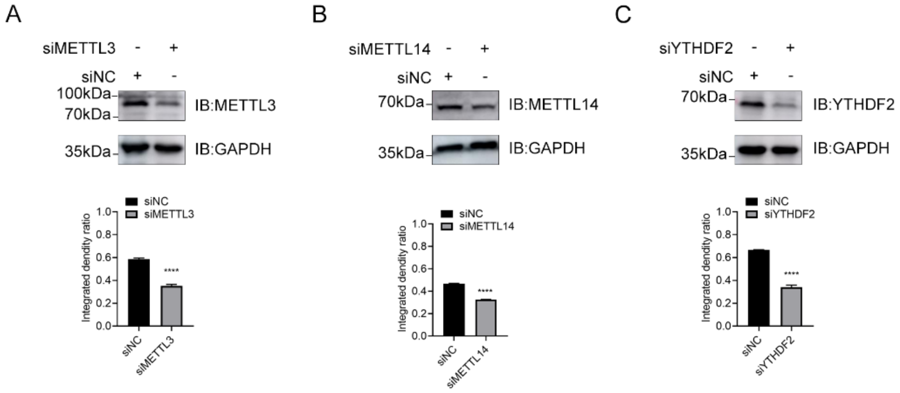

FIGS3 The relevant knockdown effect of target protein in figure 6. (A, B and C) A549 cells were transfected with siRNA targeting METTL3 (A), METTL14 (B), YTHDF2 (C) or with scrambled siRNA for 48h. Cells were harvested and protein expression levels were detected by Western-blot. The expression level of viral protein was normalized to GAPDH using ImageJ respectively. ****, $P < 0.0001$.

TableS1 Function and pathway enrichment analysis.

TableS2 Interaction of DEAD/H-box RNA helicases with influenza virus proteins

TableS3 Sequence analysis of related antiviral transcripts.
